# The mediating role of perceived spousal support in the association between psychological crisis and psychological distress among breast cancer patients and their spouses: a longitudinal study based on an actor–partner interdependence mediation model with cross-lagged analysis

**DOI:** 10.3389/fpubh.2026.1937978

**Published:** 2026-09-30

**Authors:** Xiaoli Yu, Yunfeng Ding, Zhanwei Wang, Hui Wang, Zhang Ming, Xiaofeng Ni, Yanyan Yu

**Affiliations:** Department of Breast Surgery, Huzhou Central Hospital, The Affiliated Central Hospital of Huzhou University, Huzhou, Zhejiang, China

**Keywords:** actor-partner interdependence model, breast cancer, cross-lagged analysis, longitudinal study, mediation analysis, nursing, patient, perceived spousal support

## Abstract

**Aim:**

Grounded in the Actor-Partner Interdependence Mediation Model (APIMeM), this study aimed to investigate the longitudinal dyadic interactions between psychological crisis and psychological distress among breast cancer patients and their spouses, and to examine the mediating mechanism of perceived spousal support.

**Methods:**

A convenience sample of 265 breast cancer patients and their spouses was recruited. Participants completed the Triage Assessment Form (TAF), the Distress Thermometer (DT), and the Perceived Spousal Support Scale for Breast Cancer Patients at three time points: pre-chemotherapy (T1), mid-chemotherapy (T2), and post-chemotherapy (T3). Measurement invariance testing and the Actor-Partner Interdependence Cross-Lagged Model (APIM-CLPM) were employed to identify pathways. Subsequently, an APIMeM incorporating actor-mediator-outcome and partner-mediator-outcome paths was constructed. A bias-corrected non-parametric percentile Bootstrap method (5,000 samples) was used to test the significance of the mediating effects. Missing data were handled using full information maximum likelihood (FIML) estimation.

**Results:**

After excluding 7 dropouts, data from 258 couples were analyzed. The reasons for dropout included relocation (*n* = 2), voluntary withdrawal without providing a reason (*n* = 3), and severe treatment-related complications requiring a change in treatment protocol (*n* = 2). (1) Significant actor effects were observed for both psychological crisis and distress: T1 psychological crisis positively predicted T2 psychological distress in both patients (β = 0.15) and spouses (β = 0.13, *p* < 0.01), while T1 psychological distress positively predicted T2 psychological crisis in patients (β = 0.18) and spouses (β = 0.16, *p* < 0.001). (2) Perceived spousal support significantly mediated the path from psychological crisis to psychological distress: the indirect effect was 0.06 (95% CI: 0.02–0.11) for patients and 0.05 (95% CI: 0.01–0.09) for spouses. (3) Significant partner mediating effects were found: patient T1 psychological crisis was associated with exacerbated spouse T3 psychological through the pathway of reduced spouse T2 perceived support (indirect effect = 0.04, 95% CI: 0.01–0.08).

**Conclusion:**

Perceived spousal support acts as a critical mediator linking psychological crisis to psychological distress. Clinical interventions should adopt a dyadic perspective, focusing on enhancing the quality of perceived support to interrupt the negative cycle of crisis and distress and alleviate psychological suffering in both patients and spouses.

## Introduction

Breast cancer is the most prevalent malignant tumor among women worldwide, with a persistently increasing disease burden. Globally, approximately 2.297 million new cases and 666,000 deaths occur annually in women (1). In China, there are about 357,000 new cases each year, and the median age at diagnosis is approximately 47 years (2, 3), which is younger than that in Western populations, suggesting a more prominent familial and societal impact (4, 5).

The diagnosis and comprehensive treatment of breast cancer impose not only physical trauma but also profound psychosocial repercussions. Patients commonly face fear of mortality, disturbances in body image leading to identity crises, concerns regarding diminished femininity (6), persistent anxiety over recurrence, and distress caused by treatment side effects (7, 8). Consequently, they are highly susceptible to experiencing a psychological crisis-defined as a state of psychological disequilibrium resulting from the failure of habitual coping mechanisms when confronting major life events (9). The onset of psychological crisis is a direct precursor to psychological distress, and prolonged distress is a primary driver of treatment non-adherence, self-harm, and suicidal ideation (10).

The Dyadic Coping Model, proposed by Bodenmann in the 1990s, extends the conceptualization of stress management from the individual to the couple level. It posits that when facing shared stressors, partners engage in collaborative behaviors such as communication, joint problem-solving, and emotional support. Within this framework, the quality of perceived spousal support determines whether stress crystallizes into chronic psychological distress. The model emphasizes the couple as a dynamic system; effective dyadic coping enhances relationship satisfaction and stability, making it a vital framework in chronic disease management (11–13). Spouses, serving as primary caregivers, endure substantial caregiving burdens alongside economic strain, role overload, social isolation, and profound anxiety regarding the patient’s prognosis, placing them at high risk for psychological crisis and distress (14, 15). Notably, the psychological states of patients and spouses are not isolated; they interact continuously through the marital bond. For instance, a patient’s depressive symptoms may amplify a spouse’s sense of burden, while inadequate support from the spouse may hinder the patient’s disease adaptation process (16).

However, existing research predominantly focuses on the patient in isolation or remains limited to cross-sectional correlations between partner support and patient distress, often overlooking the dynamic interactive mechanisms within the marital dyad. Specifically, there is a paucity of evidence explaining why one partner’s psychological crisis evolves into psychological distress for both individuals, particularly studies integrating perceived spousal support as a mediator within a longitudinal dyadic interaction model. To address these gaps, this study employs perceived spousal support as a mediator to construct a longitudinal Actor-Partner Interdependence Mediation Model (APIMeM). We aim to answer: (1) Does psychological crisis exacerbate psychological distress by diminishing perceived spousal support? (2) Do these mediating effects differ between actor (within-person) and partner (between-person) pathways? Our findings aim to provide empirical evidence for developing synchronous couple-based supportive interventions.

## Methods

### Participants

Using convenience sampling, 265 pairs of breast cancer patients and their spouses were recruited from the Department of Breast Surgery at our hospital between July 2024 and June 2025. Inclusion criteria for patients were: (1) meeting clinical diagnostic criteria for breast cancer (17); (2) histologically confirmed primary breast cancer; (3) undergoing initial unilateral modified radical mastectomy; (4) clinical stage I-III; (5) aged 18 years or above; and (6) possessing basic comprehension and communication abilities, with signed informed consent. Exclusion criteria included: (1) severe comorbidities affecting major organs (heart, brain); (2) serious complications; (3) withdrawal from the study; and (4) incomplete data for the three-wave longitudinal assessment. Ethical approval was obtained from the Institutional Review Board (No. 2024–06258) prior to commencement. All participants provided written informed consent.

Based on the sample size formula for repeated-measures longitudinal designs (18), considering a 10% attrition rate, a minimum of 192 dyads was required.

### Instruments

#### General information questionnaire

A self-designed questionnaire collecting demographic and clinical data (patient: age, education, residence, income, occupation, tumor stage, lesion location; spouse: age, education, residence, income).

#### Triage assessment form (TAF)

Developed by Myer et al. (19), this 3-dimensional scale (affective, cognitive, behavioral) uses a 10-point Likert scale (1 = no impairment to 10 = severe impairment). Total scores range from 3–30, with higher scores indicating greater psychological crisis severity. It was administered to both patients and spouses.

#### Distress thermometer (DT)

A single-item visual analog scale measuring psychological distress over the past week (20). Scores range from 0 (“no distress”) to 10 (“extreme distress”). A cutoff score of > = 4 indicates clinically significant distress, with reported sensitivity and specificity of 0.88 and 0.70, respectively, in Chinese cancer populations. Administered to both patients and spouses.

#### Perceived spousal support scale for breast Cancer patients

Developed by Wang Jingjing et al. (21) in China, this scale comprises 25 items across three dimensions: tangible support, informational support, and emotional support. Items are scored on a 5-point Likert scale (1 = never to 5 = always), with Item 16 reverse-scored. Total scores range from 25 to 125, with higher scores reflecting better perceived spousal support. In this study, Cronbach’s alpha ranged from 0.931 to 0.958 across time points. Composite Reliability (CR) ranged from 0.942 to 0.967, and Average Variance Extracted (AVE) ranged from 0.512 to 0.603, indicating robust psychometric properties. To facilitate dyadic analysis, spouses completed the scale with instructions modified to: “Please rate the support you perceive receiving from your partner (the patient).”

### Procedure

Research assistants were trained prior to data collection. Questionnaires were administered after obtaining hospital and participant consent. General information was extracted from medical records. Data on psychological crisis, distress, and support perception were collected at three time points: T1 (post-surgery/pre-chemotherapy), T2 (mid-chemotherapy: after the 3rd cycle for 4–6 cycle regimens, or after the 4th cycle for 8-cycle regimens), and T3 (post-chemotherapy). Surveys were conducted via face-to-face interviews in a private, confidential setting. Participants were assured anonymity and that data would be used solely for research purposes. For participants with low literacy, investigators read items aloud, and participants made independent selections. Additionally, lie scale items were incorporated into the questionnaire to ensure its reliability and verify that the responses genuinely reflected the patients’ true intentions. To maintain high participation rates, we adopted the following strategies: conducting face-to-face surveys in a confidential environment, assuring anonymity, having the investigators read the items aloud to participants with lower literacy levels, incorporating lie scale items, and providing small gifts upon completion of the questionnaire. These measures likely contributed to minimizing the attrition rate (see Figure 1).

**Figure 1:**
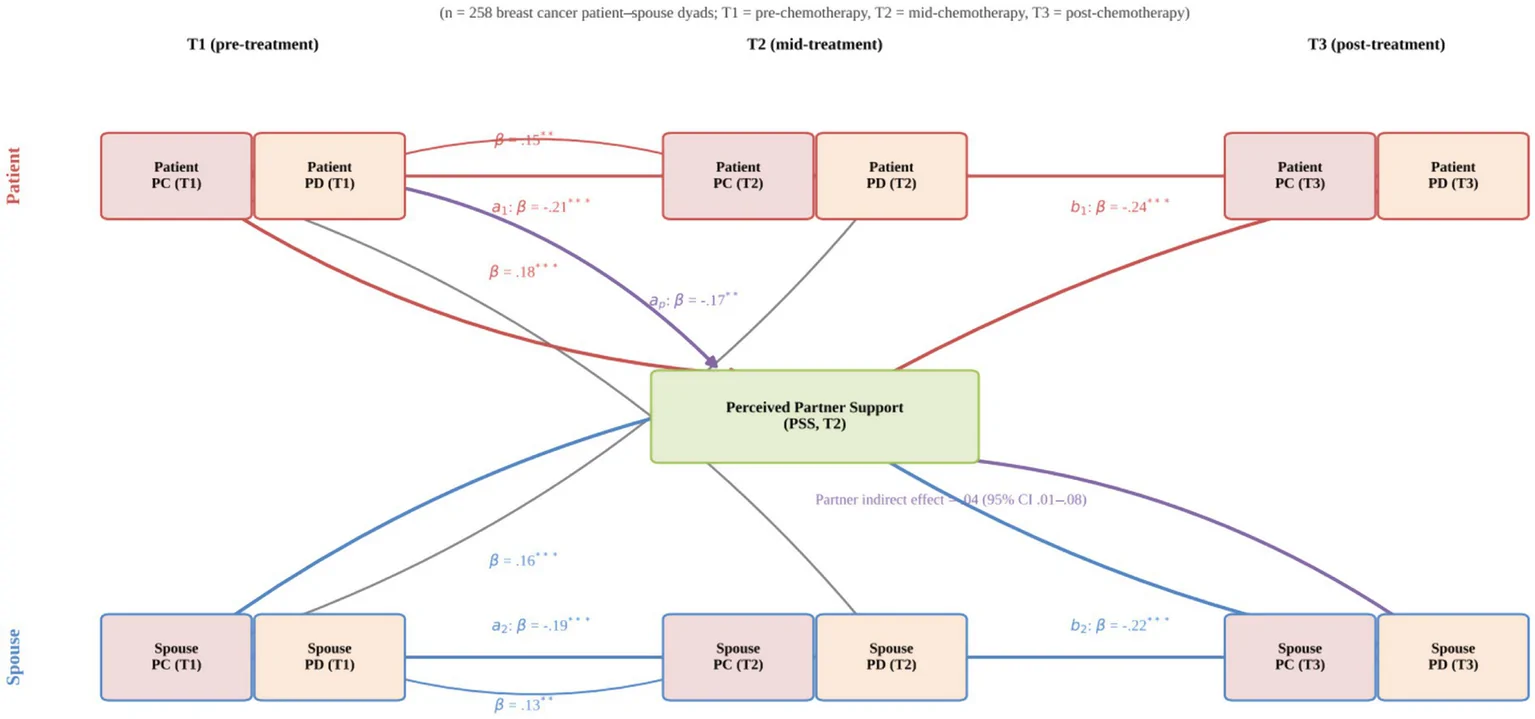
Actor–Partner interdependence mediation model (APIMeM) of perceived spousal support in the association between psychological crisis and psychological distress among breast cancer patients and their spouses.

### Statistical analysis

Data were double-entered and verified using SPSS 26.0 for descriptive statistics and correlation analyses. Mplus 8.3 was utilized for advanced modeling:

(1) Measurement Invariance Testing: Configural, metric (factor loadings), and scalar (intercepts) invariance models were tested sequentially to ensure comparability of constructs across time and between patients and spouses.
(2) Actor-Partner Interdependence Cross-Lagged Model (APIM-CLPM): Employed to examine bidirectional predictive relationships between psychological crisis and distress.
(3) Actor-Partner Interdependence Mediation Model (APIMeM): Constructed with T1 psychological crisis as the independent variable, T2 perceived spousal support as the mediator, and T3 psychological distress as the dependent variable. The model included actor and partner mediation paths while controlling for autoregressive effects and demographic covariates. Additionally, to account for the variation in T2 assessment timing across different chemotherapy regimens, the type of chemotherapy regimen (categorized as 4–6 cycle regimens vs. 8-cycle regimens) was included as a covariate in all models.
(4) Mediation Effect Testing: Bias-corrected Bootstrap estimation with 5,000 resamples was performed. Indirect effects were considered significant if the 95% confidence interval did not include zero (22).
(5) Handling of Missing Data: Full information maximum likelihood (FIML) estimation was employed under the missing-at-random (MAR) assumption to handle any item-level missing data from the 258 dyads who completed at least two waves of assessment. This approach utilizes all available data points to generate unbiased parameter estimates.

## Results

### Participants’ general characteristics

After excluding 7 dropouts, 258 valid dyads were included. The reasons for the 7 dropouts were as follows: 2 dyads relocated to other cities and could not complete follow-up; 3 dyads voluntarily withdrew from the study without providing a specific reason; and 2 dyads were excluded because the patient developed severe treatment-related complications requiring a change in the original chemotherapy protocol, rendering their subsequent data incomparable. Patient age ranged 22–78 years (mean 52.37+/−10.24); spouse age ranged 24–81 years (mean 54.12+/−10.85). Most patients were diagnosed at stage I-II (77.91%), with slightly more left-sided than right-sided lesions (55.04% vs. 44.96%). Detailed demographic information is presented in Table 1.

**Table 1:** Demographic characteristics of participants (*n* = 258).

Patient information	Category	*n*	%	Patient information	Category	*n*	%
Age (years)	<45	75	29.07	Occupation	Enterprise/Inst.	52	20.16
	45–59	82	31.78		Self-employed	22	8.53
	> = 60	101	39.15		Employee/Staff	157	60.85
Education	Junior high and below	118	45.74		Unemployed	27	10.47
	High school	77	29.84	Tumor stage	I	106	41.09
	College and above	63	24.42		II	95	36.82
Residence	Urban	133	51.55		III	57	22.09
	Rural	125	48.45	Lesion Site	Left breast	142	55.04
Income (CNY)	<3,000	45	17.44		Right breast	116	44.96
	3,000-	69	26.74				
	6,000-	94	36.43				
	>10,000	50	19.38				

Spouse information	Category	*n*	%	Spouse information	Category	*n*	%
Age (years)	<45	74	28.68	Education	Junior high and below	102	39.53
	45–59	81	31.40		High school	86	33.33
	> = 60	103	39.92		College and above	70	27.13
Income (CNY)	<3,000	32	12.40	Residence	Urban	133	51.55
	3,000-	55	21.32		Rural	125	48.45
	6,000-	114	44.19				
	>10,000	57	22.09				

### Common method bias testing

Harman’s single-factor test was employed to assess common method bias. As this was a longitudinal design, data from all three measurement waves were tested independently. The results showed that the variance explained by the first factor was 15.27, 19.64, and 23.95% for T1, T2, and T3, respectively, all below the critical threshold of 40% (23), indicating no serious common method bias in this study.

### Descriptive statistics and trends over time

Repeated-measures ANOVA revealed significant time effects for all variables (Table 2):

Psychological Crisis: Significant time effect for both patients (F = 78.62, p < 0.001) and spouses (F = 54.28, p < 0.001). Both patients and spouses showed a pattern of initial increase followed by a peak at T2 (mid-chemotherapy).
Psychological Distress: Significant time effect for both patients (F = 20.35, p < 0.001) and spouses (F = 25.68, p < 0.001). Distress levels increased progressively across the treatment course, with T3 scores higher than T1 and T2.
Perceived Spousal Support: Significant time effect for both patients (F = 32.17, p < 0.001) and spouses (F = 28.94, p < 0.001). Perceived support showed a declining trend over time, with T2 and T3 scores significantly lower than T1, suggesting that mid-chemotherapy represents a vulnerable window for support perception.

**Table 2:** Scores of psychological crisis, psychological distress, and perceived spousal support in breast cancer patients and spouses (*n* = 258, mean ± SD).

Item	T1	T2	T3	*F*-value	*p*-value
Patient psychological crisis	14.51 ± 2.43	17.31 ± 2.41	16.21 ± 2.23	78.62	<0.001
Spouse psychological crisis	11.17 ± 2.23	13.77 ± 2.43	12.24 ± 2.59	54.28	<0.001
Patient psychological distress	5.57 ± 1.21	6.03 ± 1.38	6.20 ± 1.11	20.35	<0.001
Spouse psychological distress	5.05 ± 1.17	5.22 ± 1.10	5.86 ± 1.24	25.68	<0.001
Patient perceived spousal support	92.37 ± 12.45	87.62 ± 13.81	85.14 ± 14.22	32.17	<0.001
Spouse perceived partner support	89.53 ± 13.67	84.28 ± 14.95	82.06 ± 15.38	28.94	<0.001

### Correlation analysis

Pearson correlation analysis revealed that psychological crisis, psychological distress, and perceived spousal support were significantly correlated across all time points. Specifically: psychological crisis was significantly positively correlated with psychological distress (r = 0.29–0.56, *p* < 0.01); perceived spousal support was significantly negatively correlated with both psychological crisis and psychological distress (r = −0.28 to −0.47, *p* < 0.01); and cross-partner correlations between support perception and distress were also significant, providing a foundation for subsequent mediation modeling, The correlation analysis is shown in Table 3.

**Table 3:** Correlation matrix of psychological crisis, psychological distress, and perceived spousal support among breast cancer patients and spouses (*r* values, *n* = 258).

Variable	①	②	③	④	⑤	⑥	⑦	⑧	⑨	⑩	⑪	⑫	⑬	⑭	⑮	⑯	⑰	⑱
① Patient PC T1	1																	
② Patient PC T2	0.61**	1																
③ Patient PC T3	0.53**	0.53**	1															
④ Spouse PC T1	0.55**	0.46**	0.32*	1														
⑤ Spouse PC T2	0.45**	0.51**	0.37**	0.53**	1													
⑥ Spouse PC T3	0.41**	0.47**	0.43**	0.50**	0.42**	1												
⑦ Patient PD T1	0.56**	0.48**	0.32*	0.30*	0.31*	0.30*	1											
⑧ Patient PD T2	0.50**	0.49**	0.37**	0.35**	0.38**	0.34**	0.56**	1										
⑨ Patient PD T3	0.41**	0.43**	0.46**	0.40**	0.35**	0.45**	0.44**	0.52**	1									
⑩ Spouse PD T1	0.50**	0.42**	0.31*	0.31*	0.40**	0.29*	0.49**	0.47**	0.29*	1								
⑪ Spouse PD T2	0.44**	0.45**	0.35**	0.37**	0.42**	0.32*	0.41**	0.49**	0.31*	0.46**	1							
⑫ Spouse PD T3	0.38**	0.41**	0.41**	0.42**	0.44**	0.41**	0.40**	0.40**	0.36**	0.40**	0.41**	1						
⑬ Patient PSS T1	−0.42**	−0.39**	−0.33**	−0.28**	−0.26**	−0.24**	−0.45**	−0.41**	−0.36**	−0.38**	−0.35**	−0.31*	1					
⑭ Patient PSS T2	−0.38**	−0.47**	−0.40**	−0.30*	−0.35**	−0.31*	−0.41**	−0.46**	−0.39**	−0.36**	−0.40**	−0.34**	0.61**	1				
⑮ Patient PSS T3	−0.35**	−0.41**	−0.47**	−0.27*	−0.32*	−0.38**	−0.37**	−0.42**	−0.44**	−0.33*	−0.36**	−0.40**	0.52**	0.63**	1			
⑯ Spouse PSS T1	−0.36**	−0.32*	−0.28**	−0.41**	−0.37**	−0.30*	−0.34**	−0.31*	−0.29*	−0.43**	−0.39**	−0.35**	0.45**	0.42**	0.39**	1		
⑰ Spouse PSS T2	−0.33**	−0.36**	−0.31*	−0.38**	−0.46**	−0.34**	−0.31*	−0.35**	−0.30*	−0.40**	−0.44**	−0.38**	0.42**	0.58**	0.43**	0.58**	1	
⑱ Spouse PSS T3	−0.30*	−0.34**	−0.37**	−0.35**	−0.40**	−0.41**	−0.29*	−0.33*	−0.36**	−0.36**	−0.38**	−0.44**	0.39**	0.46**	0.47**	0.51**	0.62**	1

PC, Psychological Crisis; PD, Psychological Distress; PSS, Perceived Spousal Support. *p < 0.05. **p < 0.01.

### Measurement invariance testing

Configural, metric, and scalar invariance models were constructed for psychological crisis, psychological distress, and perceived spousal support for both patients and spouses (Tables 4, 5). Model comparisons showed that ΔCFI for psychological crisis and distress was <=0.01, satisfying cross-subject measurement equivalence. For perceived spousal support, ΔCFI ranged from 0.002 to 0.006, also meeting the criteria for cross-time and cross-subject measurement invariance, indicating that subsequent dyadic data analysis was comparable.

**Table 4:** Measurement invariance model fit for psychological crisis and perceived spousal support.

Variable	Model	*χ* ^2^	df	CFI	ΔCFI	TLI	RMSEA	SRMR
Patient PC	Configural	186.30	68	0.991	–	0.983	0.038	0.023
	Metric	198.54	74	0.990	0.001	0.985	0.036	0.025
	Scalar	226.36	80	0.985	0.005	0.977	0.041	0.026
Spouse PC	Configural	192.37	68	0.990	–	0.982	0.038	0.031
	Metric	206.68	74	0.988	0.002	0.984	0.037	0.032
	Scalar	252.71	80	0.984	0.004	0.980	0.038	0.034
Patient PSS	Configural	312.45	102	0.972	–	0.961	0.042	0.028
	Metric	328.17	110	0.970	0.002	0.963	0.041	0.030
	Scalar	362.84	124	0.966	0.004	0.960	0.043	0.033
Spouse PSS	Configural	298.63	102	0.975	–	0.964	0.040	0.031
	Metric	312.59	110	0.973	0.002	0.966	0.039	0.033
	Scalar	348.72	124	0.969	0.004	0.962	0.041	0.035

**Table 5:** Measurement invariance model fit for psychological distress.

Variable	Model	*χ* ^2^	df	CFI	ΔCFI	TLI	RMSEA	SRMR
Patient PD	Configural	236.58	98	0.974	–	0.962	0.049	0.036
	Metric	244.83	106	0.973	0.001	0.966	0.048	0.037
	Scalar	295.47	118	0.971	0.002	0.964	0.047	0.037
Spouse PD	Configural	326.53	98	0.974	–	0.956	0.054	0.054
	Metric	337.19	106	0.970	0.004	0.960	0.052	0.055
	Scalar	398.08	118	0.966	0.004	0.958	0.053	0.055

### Actor-partner interdependence cross-lagged model (CLPM) analysis

An APIM-CLPM was constructed for patients and spouses (M1), and parameter constraints were applied (M2). Model comparison showed that although the Δ*χ*^2^ between M2 and M1 was 113.91 (Δdf = 38, *p* < 0.001), the ΔCFI was merely 0.001 (< 0.01), indicating that the constraints did not lead to a significant deterioration in model fit. Based on the principle of parsimony and the ΔCFI criterion, M2 was selected as the final model (Table 6).

**Table 6:** Model fit results of the actor-partner interdependence cross-lagged model.

Model	*χ* ^2^	df	CFI	ΔCFI	TLI	RMSEA	SRMR
M1 (Saturated Model)	1648.24	682	0.946	–	0.936	0.041	0.075
M2 (Constrained Model)	1762.15	720	0.945	0.001	0.937	0.04	0.076

Path analysis results showed:

Autoregressive effects were significant: All autoregressive coefficients ranged from 0.37 to 0.51 (*p* < 0.001), indicating high temporal stability for psychological crisis, psychological distress, and perceived spousal support.
Actor effects were significant: Psychological crisis to psychological distress: patients (T1 to T2: β = 0.15, T2 to T3: β = 0.13, *p* < 0.01); spouses (T1 to T2: β = 0.13, T2 to T3: β = 0.11, *p* < 0.01). Psychological distress to psychological crisis: patients (T1 to T2: β = 0.18, T2 to T3: β = 0.15, *p* < 0.001); spouses (T1 to T2: β = 0.16, T2 to T3: β = 0.14, *p* < 0.01).
Partner effects were significant: Patient psychological distress to spouse psychological distress (T1 to T2: β = 0.13, T2 to T3: β = 0.11, *p* < 0.001); spouse psychological distress to patient psychological distress (T1 to T2: β = 0.11, T2 to T3: β = 0.10, *p* < 0.01).

### Actor-partner interdependence mediation model (APIMeM) analysis

#### Model fit

The APIMeM was constructed with T1 psychological crisis as the independent variable, T2 perceived spousal support as the mediator, and T3 psychological distress as the dependent variable, controlling for autoregressive effects and demographic covariates, and the type of chemotherapy regimen. The model demonstrated good fit: *χ*^2^/df = 2.18, CFI = 0.947, TLI = 0.939, RMSEA = 0.038, SRMR = 0.071, meeting standard model fit criteria.

#### Mediation effect decomposition

Bootstrap mediation effect results are presented in Table 7.

Actor Mediation Effects: Patient T1 psychological crisis was associated with reduced T2 perceived spousal support, which was in turn associated with exacerbated T3 psychological distress (effect = 0.06, 95% CI: 0.02–0.11), accounting for 18.2% of the total effect. The spouse path showed a consistent pattern (effect = 0.05, 95% CI: 0.01–0.09), accounting for 16.7% of the total effect.
Partner Mediation Effects: Patient T1 psychological crisis was associated with reduced spouse T2 perceived support, which was in turn associated with exacerbated spouse T3 psychological distress (effect = 0.04, 95% CI: 0.01–0.08). Similarly, spouse psychological crisis showed an indirect association with exacerbated patient distress through reduced patient support perception (effect = 0.03, 95% CI: 0.01–0.07).
Total Mediation Effect: The total indirect effect of perceived spousal support was 0.18 (95% CI: 0.11–0.26), confirming that support perception is a key mechanism linking crisis to distress.

**Table 7:** Decomposition of mediation effects of perceived spousal support (95% CI).

Path	Effect	Boot SE	95% CI	Significant
Actor mediation effects				
Patient T1 PC → T2 Patient PSS → T3 Patient PD	0.06	0.02	[0.02, 0.11]	Yes
Spouse T1 PC → T2 Spouse PSS → T3 Spouse PD	0.05	0.02	[0.01, 0.09]	Yes
Partner mediation effects				
Patient T1 PC → T2 Spouse PSS → T3 Spouse PD	0.04	0.02	[0.01, 0.08]	Yes
Spouse T1 PC → T2 Patient PSS → T3 Patient PD	0.03	0.01	[0.01, 0.07]	Yes
Total mediation effect	0.18	0.04	[0.11, 0.26]	Yes

#### Supplementary analysis: support subdimensions

To further elucidate the differential roles of support types, perceived spousal support was decomposed into three subdimensions-emotional support, informational support, and tangible support-for parallel mediation analysis. The results showed that emotional support demonstrated the strongest mediating effect (patient path: β = 0.04; spouse path: β = 0.03, *p* < 0.01), while informational and tangible support showed weaker and non-significant effects (*p* > 0.05). This suggests that emotional support perception plays a dominant role in interrupting the transformation from psychological crisis to psychological distress, for details, see Table 8.

**Table 8:** Decomposition of the mediating effects of perceived partner support dimensions between psychological crisis and psychological distress (95% CI).

Path	Effect value	Boot SE	95% CI	Significant
Emotional support				
Patient T1 PC → T2 Patient Emotional Support → T3 Patient PD	0.04	0.01	[0.02, 0.06]	Yes
Partner T1 PC → T2 Partner Emotional Support → T3 Partner PD	0.03	0.01	[0.01, 0.05]	Yes
Informational support				
Patient T1 PC → T2 Patient Informational Support → T3 Patient PD	0.01	0.01	[−0.01, 0.03]	No
Partner T1 PC → T2 Partner Informational Support → T3 Partner PD	0.01	0.01	[−0.01, 0.03]	No
Tangible support				
Patient T1 PC → T2 Patient Tangible Support → T3 Patient PD	0.01	0.01	[−0.01, 0.03]	No
Partner T1 PC → T2 Partner Tangible Support → T3 Partner PD	0.01	0.01	[−0.01, 0.03]	No

## Discussion

This study, for the first time within a breast cancer patient-spouse dyadic system, employed a three-wave longitudinal design and the Actor-Partner Interdependence Mediation Model (APIMeM) to reveal the critical mediating mechanism of perceived spousal support in the transformation of psychological crisis into psychological distress. The findings not only corroborate the bidirectional longitudinal predictive relationship between psychological crisis and distress but also, more importantly, provide evidence consistent with the existence of a cross-individual transmission pathway: crisis to diminished support perception to exacerbated distress.

### Bidirectional reinforcement between psychological crisis and distress: a dyadic perspective

The cross-lagged analysis revealed significant actor and partner effects for both psychological crisis and distress, consistent with previous dyadic coping research (24), while further clarifying the temporal lag characteristics. Notably, the path coefficients from psychological crisis to distress (β = 0.13–0.15) were slightly smaller than those from distress to crisis (β = 0.14–0.18), suggesting that during the chemotherapy process, psychological distress may be a more stable precursor to subsequent crisis (25, 26). Furthermore, the presence of partner effects indicates that couples are not isolated individuals but rather a “stress community” that shares an emotional burden. The helplessness arising from treatment side effects may directly contribute to the spouse’s psychological distress through emotional contagion and declining interaction quality. This finding reaffirms the necessity of formally integrating spouses into the tumor psychological support system.

Yang et al. (27) noted that significant actor-partner interdependence effects exist between patients and spouses, wherein one party’s disease communication difficulties and negative emotions are transmitted and may exacerbate the other party’s psychological distress, forming a negative cycle. This suggests that clinical practice should shift from the traditional patient-centered model to a systemic intervention model that incorporates the couple or family unit. Clinical efforts should be devoted to constructing dual-track communication intervention strategies-including synchronous cognitive therapy, partner emotional expression training, and regular family conferences-to promote open and honest communication regarding disease-related emotions and concerns, thereby breaking down the barrier of protective buffering and enhancing dyadic coping capacity (28, 29). Family systems theory posits that family members dynamically influence one another, which may account for the lagged nature of partner effects (30). Emotional transmission renders one party’s persistent negative emotions a direct stressor for the other, triggering similar emotional disturbances; alterations in intimate interaction patterns-such as a patient’s social withdrawal or reduced emotional expression in response to distress-undermine effective communication and support efficacy between spouses, intensifying the spouse’s sense of isolation, while the caregiver burnout and maladaptive communication patterns stemming from caregiving burdens feedback into and compound the patient’s uncertainty and psychological burden (31, 32). Monitoring partner communication quality during critical chemotherapy periods and providing timely professional guidance are of critical importance for reducing patient depression risk and enhancing treatment adherence and quality of life.

### The mediating role of perceived spousal support: from correlation to mechanism

The core contribution of this study lies in confirming the mediating role of perceived spousal support between psychological crisis and distress, with this mediation operating simultaneously through both actor and partner pathways.

First, Actor Mediation Effects were significant: Patient T1 psychological crisis was associated with reduced T2 perceived spousal support, which was in turn associated with exacerbated T3 psychological distress (indirect effect = 0.06, 95% CI: 0.02–0.11). This result supports the “stress-support-adaptation” model (17) and further elucidates the psychological mechanism of Support Perception Bias. When an individual is in a state of psychological crisis, cognitive resources are occupied by negative emotions, rendering them prone to “support blindness”-even when the spouse objectively provides emotional comfort and practical care, the patient still subjectively underestimates the availability and quality of support (33). This bias weakens the buffering efficacy of social support, preventing acute psychological crisis from being promptly resolved and ultimately crystallizing into persistent psychological distress (34). The spouse path exhibited a consistent pattern (indirect effect = 0.05, 95% CI: 0.01–0.09), indicating that caregivers are similarly vulnerable to support perception deficits; their prolonged suppression of personal needs to maintain family functioning often leads to desensitization toward positive feedback (e.g., gratitude, intimate interactions) from the patient (35). In the context of Chinese culture, where familism is deeply rooted, spouses often assume the role of the ‘pillar of strength,’ habitually suppressing their own emotional needs to keep the family functioning. These traditional gender role expectations may lead to a blunted perception of positive feedback from the patient—a phenomenon we refer to as ‘support blindness.’ Concurrently, patients’ guilt over potentially burdening their family may also hinder them from proactively seeking or perceiving support. Therefore, interventions must take cultural factors into account, encouraging a break from the ‘silent caregiver’ stereotype and fostering bidirectional emotional expression.

Second, the identification of Partner Mediation Effects carries significant theoretical innovation: Patient T1 psychological crisis was associated with exacerbated spouse T3 psychological through the pathway of reduced spouse T2 perceived support (indirect effect = 0.04, 95% CI: 0.01–0.08). This finding transcends the simplistic explanation of emotional “contagion,” demonstrating that emotional transmission between spouses is not direct but rather occurs via the cognitive bridge of “support interaction quality” (36, 37). Specifically, a patient’s psychological crisis-often accompanied by irritability, withdrawal, or excessive dependence-may be interpreted by the spouse as “my support is ineffective” or “I am being rejected,” thereby diminishing the spouse’s motivation to provide support and degrading their support perception, ultimately backfiring on their own mental health. This “crisis to support negation to spouse distress” spillover effect explains why, even after a patient’s distress level declines, the spouse’s distress may persist (38, 39).

### Mid-chemotherapy: a vulnerable window for support perception

This study found that perceived spousal support scores reached their lowest point at T2 (mid-chemotherapy), coinciding with the peak of psychological crisis. Mid-chemotherapy represents a pivotal turning point connecting acute stress to chronic adaptation. At this juncture, patients face the concentrated onset of side effects such as alopecia, nausea, and vomiting, while spouses’ caregiving fatigue and emotional exhaustion also reach their zenith (40). In a state of mutual physical and psychological depletion, communication breakdowns and support misalignment are highly likely to occur (41). Therefore, T2 is not only a period of high incidence for psychological distress but also a critical period of heightened risk for support perception rupture (42). Clinical interventions that precisely target this time window with tailored support enhancement training hold promise for effectively interrupting the mediation pathway. The mediating effect of emotional support was the strongest, while the mediating effects of informational and tangible support were weaker and non-significant, suggesting that perceived emotional support plays a dominant role in blocking the transformation of psychological crisis into psychological distress. The predominant mediating effect of emotional support may be because a psychological crisis is essentially a state of emotional imbalance, during which individuals have the most urgent need for emotional connection and empathic understanding. When patients or spouses are in crisis, the lack of emotional support is strongly associated with exacerbated feelings of loneliness and helplessness, which in turn is associated with persistent psychological distress. In contrast, informational and tangible support primarily act on the cognitive and behavioral aspects of disease management, which may be masked by negative emotions during an acute crisis, leading to perceptual biases. It is recommended to conduct synchronous couple-based emotional support training during critical phases of chemotherapy (e.g., T2), focusing on guiding both partners in emotional expression and empathic listening, rather than merely providing disease-related information or daily care. Clinical nurses can teach “emotional validation” techniques (e.g., reflecting the other’s feelings, expressing understanding) to help couples establish emotional connections during crises, thereby blocking the vicious cycle of “crisis → lack of perceived support → exacerbated distress.” Concurrently, attention should be paid to the emotional exhaustion of spouses to prevent them from neglecting their own emotional needs due to long-term caregiving, thus truly realizing a psychological support model of “couples fighting together.”

### Clinical implications: constructing a “crisis-support-distress” ternary intervention model

Based on the foregoing findings, the traditional single-patient psychological intervention model urgently needs to transition toward a synchronous couple-based intervention model. The following ternary intervention strategy is proposed:

Proactive Screening: Assess couple psychological crisis levels and support perception baselines at T1 (pre-chemotherapy) to identify high-risk dyads (e.g., those with extremely low support perception or high crisis levels).
Support Empowerment: Conduct joint couple counseling sessions at T2 (mid-chemotherapy), focusing not on eliminating negative emotions but on correcting support perception biases. “Support behavior specification training” (e.g., guiding patients to articulate needs explicitly and guiding spouses to practice active listening) and “gratitude expression exercises” may be employed to enhance both parties’ awareness of and acceptance toward each other’s contributions.
Dynamic Monitoring: Incorporate perceived spousal support into routine nursing assessment indicators, particularly during mid-chemotherapy, to promptly identify early signals of support deficits and provide individualized guidance.

### Study limitations and future directions

Despite the use of a rigorous longitudinal design and advanced APIMeM statistical methods, this study has several limitations. First, the sample was drawn from a single center and only included stage I–III patients who underwent unilateral modified radical mastectomy, excluding those who underwent breast-conserving surgery, breast reconstruction, or had stage IV metastatic breast cancer. This may limit the generalizability of the findings to other breast cancer populations (e.g., advanced-stage patients or those receiving different surgical approaches). All participants were recruited from Huzhou Central Hospital, located in a region with an above-average economic level; thus, the social support resources and healthcare accessibility of this population may differ from those in other regions, which could affect the universality of the results. Future multi-center studies are needed to validate these findings. Second, perceived partner support was measured only via self-report scales; future research could incorporate behavioral observation methods (e.g., supportive conversation coding) for multimodal assessment. Third, there were discrepancies in the T2 assessment time points across different chemotherapy regimens (after the 3rd cycle for patients receiving 4–6 cycles, and after the 4th cycle for those receiving 8 cycles). Although we controlled for this as a covariate, it may still have some impact on longitudinal comparisons, and future studies should further standardize assessment time points. Although we attempted to control for this variability by including the type of chemotherapy regimen as a covariate in our statistical models, future studies with more uniform assessment schedules are warranted. Fourth, this study did not differentiate between specific types of support; although supplementary analyses suggested that emotional support had the strongest effect, future research should further explore the differential contributions of various support dimensions (emotional, informational, and tangible) within the mediation pathways. Finally, although three-wave data are superior to cross-sectional studies, they still cannot fully establish causal precedence. Future research could combine randomized controlled trials (RCTs) to verify the effectiveness of support interventions in blocking the mediating pathways.

## Conclusion

In conclusion, psychological crisis in breast cancer patients and their spouses is not only directly associated with psychological distress in both themselves and their partners but also shows a significant indirect association by diminishing perceived spousal support. Perceived spousal support serves as a dual actor-partner mediator between psychological crisis and psychological distress. Clinical nursing care should adopt a dyadic perspective, positioning the enhancement of perceived spousal support as a key intervention target for interrupting psychological crisis progression and reducing the overall level of psychological distress, thereby achieving the synergistic development of psychological well-being in both patients and spouses.

## Data Availability

The original contributions presented in the study are included in the article/supplementary material, further inquiries can be directed to the corresponding author.
